# Limits of life and ethical imperatives: A synthesis between principlism and moral deliberation in periviability

**DOI:** 10.1016/j.clinsp.2026.101031

**Published:** 2026-07-03

**Authors:** Fábio Roberto Cabar

**Affiliations:** Hospital das Clínicas da Faculdade de Medicina da Universidade de São Paulo, São Paulo, SP, Brazil

## Introduction

The Shifting Frontier and the Crisis of Uncertainty Contemporary neonatology has witnessed unprecedented technological victories, pushing the frontier of survival into what the literature terms the “gray zone” or periviability, conventionally situated between 22 weeks and 0 days and 25 weeks and 6 days of gestational age.^1^

At this threshold, viability is not a fixed biological datum, but rather a construct dependent on multiple intrinsic and extrinsic factors. Survival free of profound sequelae is the exception.^2^ This epistemological uncertainty requires medical intervention to abandon the blind technological imperative (“what can we do”) and enter the moral domain (“what ought we to do”). To address this distress, the present text synthesizes the ethical vocabulary of Beauchamp and Childress with the resolutive syntax of Diego Gracia's moral deliberation.

## The principlist tension and Gracia's hierarchy

The foundation of Anglo-Saxon clinical bioethics rests upon the four principles of Beauchamp and Childres:s^3^ Beneficence, Nonmaleficence, Autonomy, and Justice. However, in extreme periviability, these principles invariably collide. Vitalism (Sanctity of Life ‒ attempting to save at all costs in the name of Beneficence) directly clashes with the preservation of the Quality of Life (avoiding iatrogenesis and futile suffering in the name of Nonmaleficence).

To overcome the impasse of principles of “equal weight”, the model proposed by Diego Gracia Guillén^4^ establishes an ontological structure that divides the principles into two hierarchical levels:

• Level 1 (Ethics of Minimums ‒ The Public/Universal Duty): Comprises Nonmaleficence and Justice. These are absolute, coercive, and legally enforceable duties, regardless of the desires of the involved parties.
• Level 2 (Ethics of Maximums ‒ The Private/Project of Happiness): Comprises Beneficence and Autonomy. These are linked to individual life projects and family values.

Gracia postulates that the Ethics of Minimums takes absolute precedence over the Ethics of Maximums. Thus, the duty not to cause useless pain (Nonmaleficence, Level 1) to a 22-week neonate whose central nervous system is being damaged by invasive interventions supersedes the family's desire to “do everything” (Autonomy/Beneficence, Level 2). Therapeutic obstinacy is a harm (maleficence), and inflicting it in the name of another's autonomy is ethically unacceptable.

## Clinical guidelines and the dilemmas of the maternal-fetal dyad

Gracia's method of moral deliberation^5^ demands a rigorous analysis of the facts prior to valuation. International guidelines, such as those from the American College of Obstetricians and Gynecologists (ACOG), which allow for the offering of antenatal corticosteroids starting at 22-weeks provided there is an active resuscitation plan,^6^ provide these factual data. However, intervening in the periviable fetus means intervening in the woman's body, highlighting the dilemma of the maternal-fetal dyad.

Maternal Risk and the Minimums: Should the fetus experience acute distress, the fastest route of delivery (cesarean section) imposes severe morbidity on the pregnant woman and compromises her reproductive future.

Moral Deliberation: Demanding that a woman assume extremely serious physical risks to save a 22-week fetus (whose chances of intact survival are minute) constitutes a violation of maternal Nonmaleficence (Level 1). The intervention is only ethical if the woman, exercising her Ethics of Maximums (her autonomous family project), freely consents to assume this risk. Her bodily autonomy is the fundamental beacon in this decision.

## The zone of parental discretion through the lens of deliberation

In neonatology, parents act as surrogate decision-makers. While classic principlism may hypertrophy parental autonomy, Gracia's hierarchical approach finds perfect harmony with the concept of the Zone of Parental Discretion.^7^

In the Territory of Uncertainty (Ethics of Maximums): In 24-week newborns with no severe prior complications, the prognosis is truly uncertain. Nonmaleficence (Minimums) is not unequivocally threatened in the first instance. Here, the team must descend to Level 2 of deliberation: respecting parental discretion to choose between active resuscitation or palliative care, honoring their values (Autonomy and Beneficence).

In the Territory of Futility (Ethics of Minimums): If the clinical picture of the neonate (whether 22 or 25 weeks) progresses to irreversible organ failure and chronic pain, the case ascends to Level 1. The team assumes its role as the guardian of the incompetent patient's Ethics of Minimums, imposing limits on parental obstinacy through ethical and compassionate directive counseling.^8^

## Allocative justice, moral distress, and the “limit of the possible”

In developing countries, the principle of Justice and the Ethics of Minimums abandon theory and collide with a socioeconomic abyss. Intervention Bioethics requires us to view resource scarcity through the doctrine of the “reserve of the possible”.

A Neonatal ICU operates under factual limits. Justice demands systemic equity. Consuming hundreds of thousands of dollars, months of bed occupancy, and blood product supplies on a 22-week neonate with no real prospect of neurological recovery means, unappealably, denying access to term newborns with curable acute conditions.^9^

The Origin of Moral Distress: For Gracia, the attending physician should not be forced to make a “Sophie's choice” at the bedside. Deliberation regarding triage must be institutional, transparent, and supported by public health guidelines. When the system fails to do so, it generates Moral Distress among the healthcare team, forcing professionals to perform therapeutic obstinacy out of fear of litigation, thereby abandoning the universal ethical duty of Justice (Level 1) in order to protect themselves.

## Perinatal palliative care: the optimal course of action

By applying the deliberative method ‒ weighing facts, hierarchizing values, and assessing duties ‒ the bioethicist concludes that, in the face of extreme biological immaturity and technical futility, perinatal palliative care is not a “failure”, but the optimal course of action.^10^

This approach respects both of Gracia's levels simultaneously:

1. Guarantees the Ethics of Minimums: Protects the vulnerable from iatrogenesis through compassionate extubation and the abolition of dyspnea and pain with aggressive pharmacological management (Nonmaleficence), while freeing high-complexity resources for equitable use (Justice).
2. Maximizes the Possible (Ethics of Maximums): Focuses on what remains beneficent: comfort. It allows for spiritual support, skin-to-skin contact, and the building of a dignified memory for the family, respecting their residual autonomy and the dignity of grief.

## Conclusion

Care in periviability transcends biological frontiers and demands a robust ethical framework, which the union between Beauchamp, Childress, and Diego Gracia masterfully provides. Principlism gives us the map of medical virtues, but it is Gracia's hierarchy and moral deliberation that offer the compass amidst the storm of uncertainty.

Acknowledging that the public duty to not cause harm and to be just (Ethics of Minimums) precedes and limits the private desire to attempt the impossible (Ethics of Maximums) is not an act of medical surrender. On the contrary: by recognizing the factual limit of the body (viability), the system (the reserve of the possible), and science itself, the diligent transition to palliative care represents the most refined application of clinical compassion and the definitive triumph of human care over the blind technological imperative.

## Declaration of competing interest

The authors declare no conflicts of interest.

## Data Availability

There is no data to be requested.
